# Brazilian Protocol for Sexually Transmitted Infections, 2020: HIV infection in adolescents and adults

**DOI:** 10.1590/0037-8682-588-2020

**Published:** 2021-05-17

**Authors:** Lauro Ferreira da Silva Pinto, Filipe de Barros Perini, Mayra Gonçalves Aragón, Marcelo Araújo Freitas, Angélica Espinosa Miranda

**Affiliations:** 1 Escola de Ciências da Saúde da Santa Casa de Misericórdia de Vitória, Vitória, ES, Brasil.; 2 Secretaria Municipal de Saúde de Florianópolis, Florianópolis, SC, Brasil.; 3 Ministério da Saúde, Secretaria de Vigilância em Saúde, Brasília, DF, Brasil.; 4 Universidade Federal do Espírito Santo, Programa de Pós-Graduação em Doenças Infecciosas, Vitória, ES, Brasil.

**Keywords:** Clinical protocols, HIV, Acquired immunodeficiency syndrome, Drug therapy, Comorbidity

## Abstract

HIV infection is presented in the chapters of the Clinical Protocol and Therapeutic Guidelines for Comprehensive Care for People with Sexually Transmitted Infections, published by the Brazilian Ministry of Health in 2020. Health professionals and managers must learn the signs and symptoms of HIV infection and know how to diagnose it to provide appropriate treatment and reduce complications. HIV infection has become a chronic disease. Its treatment includes addressing common comorbidities such as arterial hypertension, diabetes, and dyslipidemia, in addition to cardiac risk assessment, cancer prevention, and guidance on immunization. Initiation of treatment for HIV patients is recommended regardless of clinical or immunological criteria as adopted by the Ministry of Health since 2013. Lately, it has been simplified with more tolerable first-line medications and fewer drug interactions, making its management easy to implement, including by primary health care.

## Highlighted excerpt

HIV cases are concentrated in specific population groups, such as sex workers, men who have sex with men, transexuals, people who use alcohol or other drugs, and vulnerable people, such as black, incarcerated, or people living on the streets.

## FOREWORD

This article addresses the chapter on human immunodeficiency virus, human immunodeficiency virus, HIV infection in the Clinical Protocol and Therapeutic Guidelines (PDCT) for Comprehensive Care for People with Sexually Transmitted Infections (STI) - PCDT-STI^1^ and the PDCT for Managing HIV Infections in Adults - PCDT-HIV^2^, published by the Health Surveillance Department of the Brazilian Ministry of Health. 

## EPIDEMIOLOGICAL ASPECTS

HIV is a lentivirus that causes the acquired immunodeficiency syndrome, aids, leading to an immunologic system progressive deterioration, mainly affecting CD4 T-cell macrophages, and dendritic cells^3^. The infection decreases the number of CD4+ T lymphocytes through different mechanisms, among which bystander cell apoptosis, infected cell viral death, and CD4+ T lymphocytes death through cytotoxic CD8+ T lymphocytes that recognize infected cells. When the number of CD4+ T lymphocytes is lower than the acceptance threshold, the body loses cell-mediated immunity and progressively becomes more susceptible to opportunistic infections^4^.

HIV can be transmitted through blood, semen, vaginal lubrication, or breast milk. HIV is present in those bodily fluids both as free particles and infected immunity cells^5^. The main transmission routes are unprotected sexual intercourse, contaminated syringe sharing, and mother-to-child transmission during pregnancy and breastfeeding^6^. Transmission risk through saliva is virtually none^5^.

HIV/aids epidemic in Brazil is deemed nationally stable. The HIV prevalence in the general population is 0.4%^7^. According to data from the Ministry of Health, in 2018, 43,941 new HIV cases and 37,161 aids cases were diagnosed in Brazil, with a detection rate of 17.8/100,000 inhabitants. Since 2012, an aids detection rate decrease is observed in Brazil, dropping from 21.4/100,000 inhabitants (2012) to 17.8/100,000 inhabitants in 2018, setting a 16.8% decrease. Such reduction of detection rate has been more marked after the all-case treatment recommendation, regardless of CD4+ T lymphocyte levels, implemented in December 2013^8^. HIV infection is concentrated in specific population groups, such as sex workers (5%)^9^, men who have sex with men (18%)^10^, transexuals (17%-37%)^11^, people who use alcohol or other drugs (5%)^12^, and vulnerable people, such as black, incarcerated or people living on the streets^13^.

The estimation for the end of 2018 was around 900,000 people living with HIV (PLHIV) in Brazil, among which 85% were diagnosed, 81% were associated with some health service, and 71% were kept in the services, i.e., their health was systematically followed-up in the same healthcare service. In the same period, antiretroviral therapy coverage (ART) was 66%, and viral suppression (viral load below 1,000 copies/mL) was 62% among all HIV-infected individuals^7^. Pre-exposure prophylaxis (PrEP) is available since January 2018 in the Brazilian National Health System (SUS), with more than 11,000 people enrolled by 2019^7^. The PrEP Brazil study, developed aiming to evaluate acceptance, viability, and the best form of offering PrEP to the Brazilian population as HIV prevention, showed this strategy's efficiency and viability in a real-world scenario^14^. PrEP offer in public health clinics in a middle-income environment can retain a significant number of participants and reach high adherence levels in the investigated populations without risk compensation^15^. Post-exposure prophylaxis (PEP) use has also been increasing in Brazil; the number of PEP dispensations rose from 15,540 in 2009 to 107,345 in 2018^7^.

## CLINICAL ASPECTS

The clinical manifestations of HIV infection encompass a wide range of signs and symptoms, with different phases, depending on individual immune response and viral replication intensity^16^. An acute infection picture frequently occurs in the first few weeks, followed by an asymptomatic stage, which can last years before aids emergence. In untreated individuals, the average time from HIV contamination to aids emergence is around ten years^17^ ^,^ ^18^. HIV infection can be classified into three stages:

*Acute HIV infection:* acute HIV infection is similar to other viral infections. The acute retroviral syndrome occurs between the first and third weeks of infection and is characterized by unspecific symptoms, such as fever, headache, adenopathy, pharyngitis, exanthem, and myalgia. Lymphadenomegaly afflicts mostly anterior and posterior cervical, submandibular, occipital, and axillary chains. The acute retroviral syndrome is self-limited, with spontaneous resolution within three to four weeks. In the case of an acute viral picture in a sexually active person, the doctor must consider the possibility of the acute retroviral syndrome among the differential diagnosis^19^ ^,^ ^20^.

**FIGURE 1:**
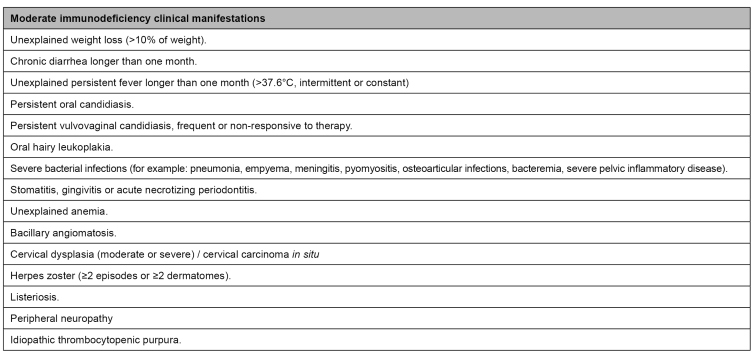
Moderate immunodeficiency clinical manifestations.

**Source:** adapted from the Clinical Protocol and Therapeutic Guidelines for Managing HIV Infections in Adults, 2018^22^.

*Clinical latency:* it is characterized as being generally asymptomatic, lasting years. It is possible to find lymphadenomegaly and unspecific laboratory test changes with low clinical significance, such as thrombocytopenia, anemia (normochromic and normocytic), and leukopenia. As the infection progresses, there is a gradual drop of CD4+ T lymphocytes, with intermittent infection emergence, which may have atypical presentations, or past infection reactivation, such as tuberculosis and herpes zoster. Besides, there can be signs and symptoms such as low-grade fever, weight loss, night sweat, fatigue, diarrhea, headache, leukoplakia, and oral candidiasis. Moderate immunodeficiency manifestations can arise in this stage (Figure 1)^21^ ^,^ ^22^.

**FIGURE 2:**
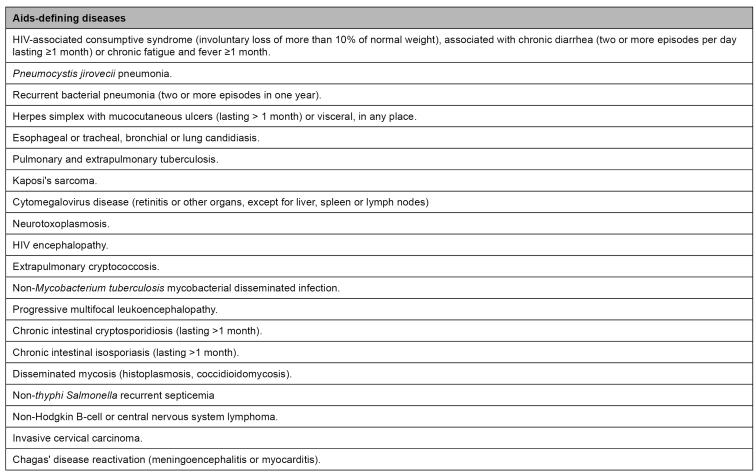
Advance immunodeficiency clinical manifestations.

**Source:** adapted from the Clinical Protocol and Therapeutic Guidelines for Managing HIV Infections in Adults, 2018^22^.

*Aids:* characterized by the emergence of advanced immunodeficiency manifestations (Figure 2)^22^. Opportunistic infections or cancer indicate aids. Depending on the immunosuppression degree and each case's specificities, there can be one or different opportunistic infections simultaneously.

## DIAGNOSIS

Adequate HIV epidemic control implies exhaustive and fast testing without coercion or discrimination. Testing is especially recommended for people with a high risk of HIV infection, including those with acute or chronic infection symptoms, people with STI and pregnant woman. Vulnerable populations, such as men who have sex with men with unknown infection status, drug users, and sex workers, should also be tested. Testing any sexually active person, especially those with a substantial risk of HIV infection, is recommended^22^ ^-^ ^24^.

**FIGURE 3:**
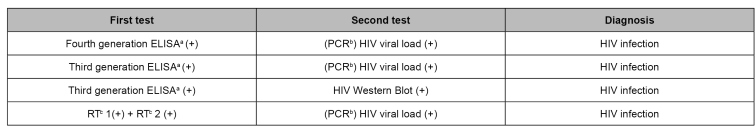
Diagnostic tests for HIV infection detection.

**Source:** adapted from the Technical Manual for HIV Infection Diagnosis, 2018^25^.

**Notes:** a) ELISA: enzyme-linked immunossorbent assay; b) PCR: polymerase chain reaction; c) RT: Rapid tests 1 and 2 from different manufacturers.

An HIV infection case is deemed as the one presenting positive results for two tests, with different methodologies^25^, of any of the four combinations described in Figure 3. In any test combination, when the first sample is negative, the person is considered noninfected, and there is no need for additional tests^25^. Third-generation rapid tests, widely available in SUS, have a 30-day window period^25^.

HIV infection diagnosis represents a unique moment in PLHIV's lives, whose reactions vary according to each individual's experiences and previous knowledge. One of the primary healthcare objectives is setting a trustworthy and respectful relationship between the healthcare professional and the patient.

## TREATMENT

Antiretroviral treatment objectives are reducing morbidity and mortality and preventing HIV transmission to other people^26^ ^,^ ^27^. Treatment must result in maximum HIV suppression to meet these goals. Therefore, treatment adherence is an essential condition for success, and it must be discussed since the first medical appointment^22^.

**FIGURE 4:**
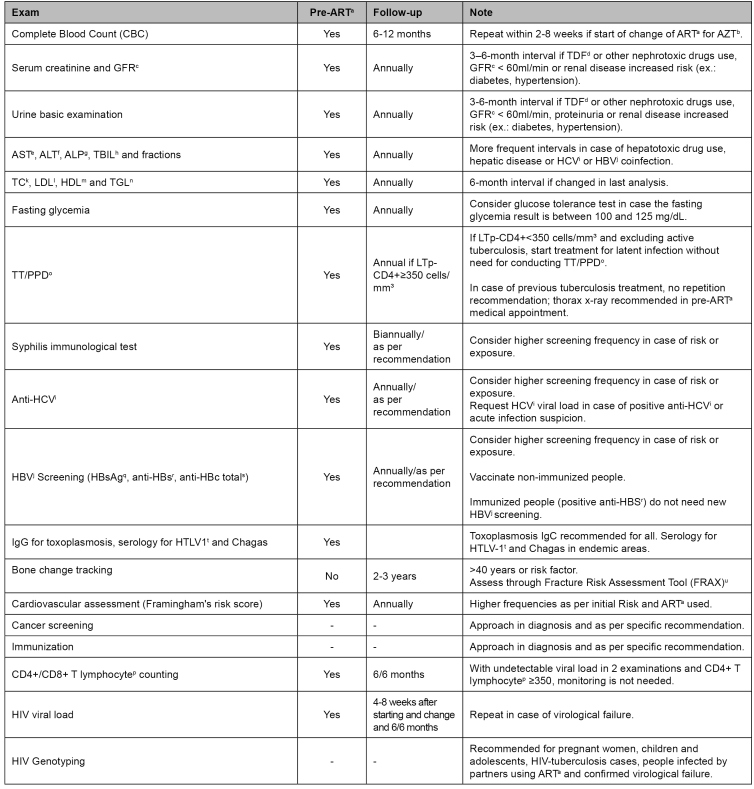
People with HIV supplementary initial and follow-up exams.

**Source:** adapted from the Clinical Protocol and Therapeutic Guidelines for Managing HIV Infections in Adults, 2018^22^.

**Notes:** a) ART: antiretroviral therapy; b) AZT: zidovudine; c) GFR: estimated glomerular filtration rate; d) TDF: tenofovir; e) AST: aspartate aminotransferase; f) ALT: alanine aminotransferase; g) ALP: alkaline phosphatase; h) TBIL: total bilirubin; i) HCV: hepatitis C virus; j) HBV: hepatitis B virus; k) TC: total cholesterol; l) LDL: low-density lipoprotein; m) HDL: high-density lipoprotein; n) TGL: triglycerides; o) TT/PPD: tuberculin test / purified protein derivative; p) LT: T lymphocyte; q) HBsAg: hepatitis B surface antigen; r) anti-HBs: hepatitis B surface antigen antibodies; s) total anti-HBc: total hepatitis B surface antigen antibodies; t) HTLV: human T-lymphotropic virus; u) Available from: https://www.sheffield.ac.uk/FRAX/tool.aspx?country=55. When using FRAX calculator, we must click on the osteoporosis secondary cause box (field "10. Secondary osteoporosis").

*HIV-infected adolescent and adult initial approach*: establishing an empathic and welcoming relationship with the infected person is necessary. Careful anamnesis must detect risk situations, STI history, chronic diseases, and immunizations. The physical examination must be complete and include skin and oral cavity detailed examination, blood pressure testing, body mass calculation, and abdominal circumference measuring. Supplementary initial and follow-up exams are described in Figure 4 ^22^.

*Antiretroviral therapy*: the ART immediate start is recommended for all PLHIV, even those asymptomatic, regardless of their clinical and immunologic stage. The initial therapy must include combinations of three antiretrovirals, with two from nucleoside or nucleotide analog reverse-transcriptase inhibitors, together with one antiretroviral from another class^22^. This other class can be of non-nucleoside or nucleotide analog reverse-transcriptase inhibitors, or protease inhibitors, reinforced with ritonavir or integrase inhibitors. Nationally in Brazil, the preferred scheme recommended for starting the treatment is lamivudine + tenofovir + dolutegravir^22^. This scheme encompasses the daily use of two pills and is highly well-accepted, with few cases of insomnia and headache^28^. Antiretrovirals must be used carefully in people treated with anticonvulsants such as phenytoin, phenobarbital, and carbamazepine, and for all tuberculosis-HIV cases coinfection, due to interaction with rifampicin^29^ ^,^ ^30^. Also, dolutegravir must not be coadministered with oxcarbazepine, dofetilide, or pilsicainide, constantly evaluating the possibility of changing such medications to enable the use of dolutegravir^29^. Another exception is represented by women of childbearing age that intend to get pregnant due to the potential risk of neural tube formation defect posed by dolutegravir in the first 12 weeks of pregnancy, despite being a very low risk (0.19%)^31^. In such case, dolutegravir must be replaced by efavirenz, a non-nucleoside analog reverse-transcriptase inhibitor, with previous genotyping, due to primary resistance risk^32^.

*Antiretroviral treatment failure:* virologic failure is characterized by HIV detectable viral load after six months from starting the treatment or therapy change, or detectable viral load in individuals under treatment if they were previously undetectable. In virologic failure, possible low treatment adherence must be investigated, as well as the presence of HIV strains with antiretroviral resistance mutations. In this case, genotyping test helps choose the rescue scheme with more efficient viral suppression^22^.

*Comorbidities in PLHIV using antiretroviral therapy:* as HIV infection has become a chronic disease, cardiovascular diseases, hypertension, diabetes, metabolic syndrome, and other comorbidities have become prevalent among PLHIV^33^ ^-^ ^35^. Smoking, dyslipidemia, renal, hepatic, osteoarticular, and cognitive alterations also need to be managed^33^ ^,^ ^36^ ^,^ ^37^. Therefore, a comprehensive approach must be conducted with such people, aligned with primary healthcare principles.

*HIV infection laboratory monitoring using CD4+ T lymphocyte counting and viral load:* CD4+ T lymphocyte counting is one of the most important examinations for assessing opportunistic immunization and prophylaxis recommendation^22^. It enables the level of immune system impairment to be evaluated, verify the recovery of an immune response to treatment, and define the moment for interrupting prophylaxis. For stable cases, in ART, with undetectable viral load and CD4+ T lymphocyte counting above than 350 cells/mm³, conducting a CD4+ T lymphocyte examination does not benefit clinical-laboratory monitoring. Laboratory and physiological CD4+ T lymphocyte fluctuations are not clinically relevant, and they can lead to conduct errors, such as early change of ART schemes or maintenance of virologic failure schemes^38^ ^-^ ^41^. The viral load must be the main focus of laboratory monitoring in PLHIV using antiretroviral therapy, enabling the early detection of virologic failure. In Brazil, healthcare professionals can refer to the Report System (*Sistema Laudo*), which provides information for the clinical monitoring of people living with HIV, such as a history of CD4+ T lymphocyte and viral load examination, history of ART dispensation, and genotyping results^42^.

*Supplementary exams and clinical follow-up assessments:* in addition to CD4+ lymphocyte counting and viral load examinations, other parameters must be monitored in PLHIV. Clinical follow-up with supplementary exams is needed. The conducted evaluations' frequency depends on clinical conditions and antiretroviral therapy use by the PLHIV (Figure 4)^22^. The importance of STI investigation, active tuberculosis, cardiovascular risk, and cancer screening (especially cervical cancer in cisgender women and transexual men) can be highlighted^43^.

Clinical follow-up must be adequate to the PLHIV's clinical conditions and treatment stage. The first return after starting or changing ART must take place around seven to 15 days, assessing adverse events and problems related to medication adherence. ART must be individually evaluated for adaptation, and monthly returns may be needed to reach greater adherence. Determining a viral load examination is recommended from four to eight weeks of treatment to assess efficiency. We recommend the minimum frequency of medical appointments as six months for stable clinical pictures using ART. In such cases, control exams are conducted biannually or according to assessment and recommendation. In the intervals between medical appointments, adherence reinforcement must be stimulated when dispensing medicines or conducting exams^22^.

**FIGURE 5:**
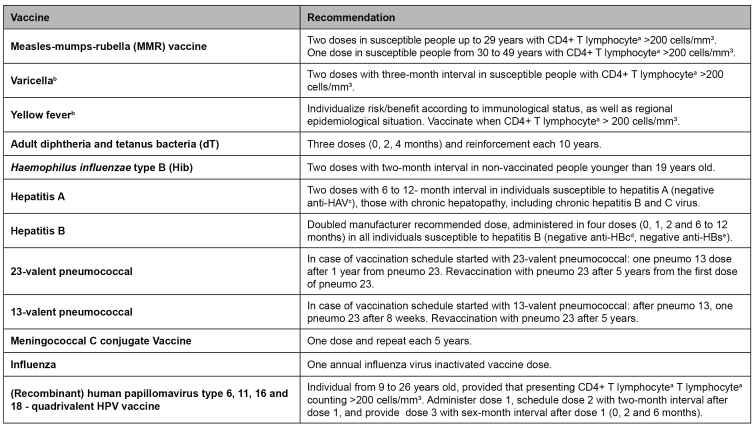
Vaccine schedule for people older than 13 years old with HIV infection.

**Source:** adapted from the Clinical Protocol and Therapeutic Guidelines for Managing HIV Infections in Adults, 2018^22^.

**Notes:** a) LT: T lymphocytes; b) Contraindication for pregnant women; c) anti-HAV: Hepatitis A antibodies; d) anti-HBc: Hepatitis B nucleus antigen antibodies; e) anti-HBs: Hepatitis B surface antigen antibodies.

*Immunization:* all vaccines in the national schedule are recommended for adults and adolescents living with HIV, provided they do not present any critical immunological deficiency. In case of symptoms or severe immunodeficiency (CD4+ T lymphocytes lower than 200 cells/mm³), it is recommended to postpone administering vaccines if possible. Live attenuated virus, and bacteria vaccines must not be administered to those with CD4+ T lymphocytes lower than 200 cells/mm³; for those with CD4+ T lymphocytes ranging from 200 and 350 cells/mm³, the risks and benefits of such vaccines must be assessed (Figure 5)^22^ ^,^ ^44^.

## SURVEILLANCE, PREVENTION, AND CONTROL

The use of accessible language by healthcare professionals is critical for PLHIV to understand the aspects of infection, transmissibility, clinical and laboratory assessment routine, treatment adherence, and stigma and discrimination overcoming^22^. Dialog allows for clarifications and helps to surpass clinical, social, and behavioral difficulties. 

Other aspects that professionals should routinely approach are the person's and their partners' sexual health and their reproductive intentions. Objective guidelines on the current combination prevention strategies help to reduce HIV and other STI transmission risk and allow for the decision on conception to be taken in the best clinical scenario, with the lowest chances of vertical and sexual transmission^45^ ^,^ ^46^.

PLHIV's sexual partners must have ethical access to timely diagnosis and treatment. For seronegative partners, it is important to offer combined prevention strategies, such as using condoms, in addition to investigating other STIs and assessing pre-exposure prophylaxis^47^.

HIV infection notification follows the same secrecy criteria defined in the Brazilian Access to Information Law ( nº 12.527/2011)^48^. Healthcare professionals must notify all HIV and people living with aids cases, even if previously reported as HIV infection. According to the Ministry of Health Ordinance^49^, defining the National Compulsory Notification List, published on June 06, 2014, people with HIV infection under clinical laboratory follow-up and previously diagnosed must be notified as they attend the healthcare service network. Private laboratories must periodically report to epidemiological surveillance all the HIV infections identified.

## SPECIAL POPULATIONS AND SITUATIONS

*Tuberculosis-HIV coinfection:* PLHIV must be screened for tuberculosis in all medical appointments. The investigation of the extrapulmonary and disseminated forms of tuberculosis is also necessary. In antiretroviral therapy cases not yet initiated, with CD4+ T lymphocytes counting lower than 50 cells/mm³, it is recommended to start treatment for tuberculosis first and introduce ART within up to two weeks. In cases of CD4+ T lymphocytes equal to or greater than 50 cells/mm³, antiretroviral therapy may be started up to the eighth week, close to the start of tuberculosis treatment maintenance stage^50^.

The initial ART for people with tuberculosis-HIV coinfection is tenofovir + lamivudine + efavirenz through pre-treatment genotyping. In case it is not possible, or if the result is not available within two weeks, dolutegravir instead of efavirenz must be used. During tuberculosis treatment and up to 15 days after its end, double the usual dose of dolutegravir must be used. If active tuberculosis is dismissed, the treatment implementation for latent infection by *Mycobacterium tuberculosis* must be assessed^22^.

*Syphilis and HIV coinfection:* syphilis treatment and diagnosis in PLHIV must be conducted the same way as in people without HIV infection. However, in PLHIV, there can be more frequent syphilis stages overlap, more symptoms, and more aggressive lesions^51^ ^,^ ^52^. It's very important investigate neurosyphilis, in the presence of neurological or ophthalmological symptoms, active tertiary syphilis or after failure of clinical treatment. If the person presents ocular and neurological signs and symptoms, they must be urgently forwarded to the specialist^51^ ^,^ ^53^.

*Populations with greater urgency to start antiretroviral therapy:* ART must start once HIV infection diagnosis is set, regardless of clinical and immunological criteria. Many people present fatal evolution without even having started treatment, despite universal access to therapy in Brazil^54^. Nonetheless, there are situations that require more urgent ART starting, such as pregnant women, due to HIV vertical transmission impact; people with severe comorbidities, such as active tuberculosis, hepatitis B or C and people with high cardiovascular risk; and cases with CD4+ T lymphocytes less than 350 cells/mm³ and those symptomatic, due to the critical impact on morbimortality^22^.

## References

[1] Brasil. Ministério da Saúde (2018). Portaria MS/SCTIE nº 42, de 5 de outubro de 2018. Torna pública a decisão de aprovar o Protocolo Clínico e Diretrizes Terapêuticas para Atenção Integral às Pessoas com Infecções Sexualmente Transmissíveis (IST), no âmbito do Sistema Único de Saúde - SUS. Diário Oficial da União.

[2] Brasil. Ministério da Saúde (2017). Portaria MS/SCTIE nº 50, de23denovembro de2017. Torna pública a decisão de atualizar o Protocolo Clínico e Diretrizes Terapêuticas para prevenção de transmissão vertical de HIV, sífilis e hepatites virais, no âmbito do Sistema Único de Saúde - SUS. Diário Oficial da União.

[3] Dullaers M, Thielemans K (2006). From pathogen to medicine: HIV-1-derived lentiviral vectors as vehicles for dendritic cell based cancer immunotherapy. J Gene Med.

[4] Fackler OT, Alcover A, Schwartz O (2007). Modulation of the immunological synapse: a key to HIV-1 pathogenesis?. Nat Rev Immunol.

[5] Shaw GM, Hunter E (2012). HIV transmission. Cold Spring Harb Perspect Med.

[6] Simon V, Ho DD, Abdool-Karim Q (2006). HIV/AIDS epidemiology, pathogenesis, prevention, and treatment. Lancet.

[7] Ministério da Saúde (BR). Secretaria de Vigilância em Saúde. Departamento de Condições Crônicas e Infecções Sexualmente Transmissíveis (2019). http://www.aids.gov.br/pt-br/pub/2019/relatorio-de-monitoramento-clinico-do-hiv-2019.

[8] Ministério da Saúde (BR). Secretaria de Vigilância em Saúde. Departamento de Condições Crônicas e Infecções Sexualmente Transmissíveis (2019). HIV/Aids 2019. BolEpidemiol.

[9] Ferreira-Júnior ODC, Guimarães MDC, Damacena GN, Almeida WDS, Souza-Júnior PRB, Szwarcwald CL, Brazilian FSW Group (2018). Prevalence estimates of HIV, syphilis, hepatitis B and C among female sex workers (FSW) in Brazil, 2016. Medicine (Baltimore).

[10] Kerr L, Kendall C, Guimarães MDC, Salani Mota R, Veras MA, Dourado I (2018). HIV prevalence among men who have sex with men in Brazil: results of the 2nd national survey using respondent-driven sampling. Medicine (Baltimore).

[11] Bastos FI, Bastos LS, Coutinho C, Toledo L, Mota JC, Velasco-de-Castro CA (2018). HIV, HCV, HBV, and syphilis among transgender women from Brazil: assessing different methods to adjust infection rates of a hard-to-reach, sparse population. Medicine (Baltimore).

[12] Bastos FI, Bertone N (2014). Pesquisa nacional sobre o uso de crack: quem são os usuários de crack e/ou similares do Brasil? Quantos são nas capitais brasileiras?.

[13] Pascom ARP, Meireles MV, Benzaken AS (2018). Sociodemographic determinants of attrition in the HIV continuum of care in Brazil in 2016. Medicine (Baltimore).

[14] PrEP Brasil Profilaxia Pré Exposição (2020). Estudo PrEP Brasil.

[15] Grinsztejn B, Hoagland B, Moreira RI, Kallas EG, Madruga JV, Goulart S (2018). Retention, engagement, and adherence to pre-exposure prophylaxis for men who have sex with men and transgender women in PrEPBrasil: 48 week results of a demonstration study. Lancet HIV.

[16] Pantaleo G, Graziosi C, Fauci AS (1993). New concepts in the immunopathogenesis of humanimmuno deficiency virus infection. N Engl J Med.

[17] Bacchetti P, Moss AR (1989). Incubation period of AIDS in San Francisco. Nature.

[18] Pedersen C, Lindhardt BO, Jensen BL, Lauritzen E, Gerstoft J, Dickmeiss E (1989). Clinical course of primary HIV infection: consequences for subsequent course of infection. BMJ.

[19] Cohen MS, Shaw GM, McMichael AJ, Haynes BF (2011). Acute HIV-1 infection. N Eng J Med.

[20] Bottone PD, Bartlett AH (2017). Diagnosing acute HIV infection. Pediatr Ann.

[21] Daar ES, Little S, Pitt J, Santangelo J, Ho P, Harawa N (2001). Diagnosis of primary HIV-1 infection. Los Angeles County Primary HIV Infection Recruitment Network. Ann Intern Med.

[22] Ministério da Saúde (BR). Secretaria de Vigilância em Saúde. Departamento de Vigilância, Prevenção e Controle das Infecções Sexualmente Transmissíveis, do HIV/Aids e das Hepatites Virais (2018). Protocolo clínico e diretrizes terapêuticas para manejo da infecção pelo HIV em adultos.

[23] Chou R, Dana T, Grusing S, Bougatsos C (2019). Screening for HIV infection in asymptomatic, nonpregnant adolescents and adults: updated evidence reportand systematic review for the US preventive services task force. JAMA.

[24] Bert F, Gualano MR, Biancone P, Brescia V, Camussi E, Martorana M (2018). Cost-effectiveness of HIV screening in high-income countries: a systematic review. Health Policy.

[25] Ministério da Saúde (BR). Secretaria de Vigilância em Saúde. Departamento de Vigilância, Prevenção e Controle das Doenças Sexualmente Transmissíveis, Aids e Hepatites Virais (2018). Manual técnico para o diagnóstico da infecção pelo HIV.

[26] Cohen MS, Chen YQ, McCauley M, Gamble T, Housseinipour MC, Kumarasamy N (2016). Antiretroviral therapy for the prevention of HIV-1 transmission. N Engl J Med.

[27] Le Messurier J, Traversy G, Varsaneux O, Weekes M, Avey MT, Niragira O (2018). Risk of sexual transmission of human immunodeficiency virus with antiretroviral therapy, suppressed viral load and condom use: a systematic review. CMAJ.

[28] Peñafiel J, Lazzari E, Padilla M, Rojas J, Gonzalez-Cordon A, Blanco JL (2017). Tolerability of integrase inhibitors in a real-life setting. J Antimicrob Chemother.

[29] Ministério da Saúde (BR). Secretaria de Vigilância em Saúde. Departamento de Condições Crônicas e Infecções Sexualmente Transmissíveis (2020). Ofício circular no 03/2020/CGAHV/DCCI/SVS/MS.

[30] Ministério da Saúde (BR). Secretaria de Vigilância em Saúde. Departamento de Condições Crônicas e Infecções Sexualmente Transmissíveis (2020). Ofício circular no 02/2020/CGAHV/DCCI/SVS/MS.

[31] Zash R, Holmes L, Diseko M, Jacobson DL, Brummel S, Mayondi B (2019). Neural-tube defects and antiretroviral treatment regimens in Botswana. N Engl J Med.

[32] Ministério da Saúde (BR). Secretaria de Vigilância em Saúde. Departamento de Condições Crônicas e Infecções Sexualmente Transmissíveis (2019). Ofício Circular no 2/2019/DCCI/SVS/MS.

[33] Wing EJ (2016). HIV and aging. Int J Infect Dis.

[34] Ghosn J, Taiwo B, Seedat S, Autran B, Katlama C (2018). HIV. Lancet.

[35] Pinto LF, Neves MB, Ribeiro-Rodrigues R, Page K, Miranda AE (2013). Dyslipidemia and fasting glucose impairment among HIV patients three years after the first antiretroviral regimen in a Brazilian AIDS outpatient clinic. Braz J Infect Dis.

[36] Pinto LF, Ragi-Eis S, Vieira NF, Soprani M, Neves MB, Ribeiro-Rodrigues R (2011). Low bone mass prevalence, therapy type, and clinical risk factors in an HIV-infected Brazilian population. J Clin Densitom.

[37] Pinto LF, Milanez MC, Golub JE, Miranda AE (2012). Malignancies in HIV/AIDS patients attending an outpatient clinic in Vitória, State of Espírito Santo, Brazil. Rev Soc Bras Med Trop.

[38] Deeks SG, Barbour JD, Martin JN, Swanson MS, Grant RM (2000). Sustained CD4+ T cell response after virologic failure of protease inhibitor-based regimens in patients with human immunodeficiency virus infection. J Infect Dis.

[39] Maggiolo F, Leone S (2010). CD4+ T lymphocyte recovery in individuals with type 1 human immunodeficiency virus infection. Clin Infect Dis.

[40] Sigaloff KC, Hamers RL, Wallis CL, Kityo C, Siwale M, Ive P (2011). Unnecessary antiretroviral treatment switches and accumulation of HIV resistance mutations; two arguments for viral load monitoring in Africa. J Acquir Immune Defic Syndr.

[41] Rawizza HE, Chaplin B, Meloni ST, Eisen G, Rao T, Sankalé JL (2011). Immunologic criteria are poor predictors of virologic outcome: implications for HIV treatment monitoring in resource- limited settings. Clin Infect Dis.

[42] Ministério da Saúde (BR). Secretaria de Vigilância em Saúde. Departamento de Vigilância, Prevenção e Controle das IST, do HIV/Aids e das Hepatites Virais (2018). Sistema laudo.

[43] Casper C, Crane H, Menon M, Money D, Holmes KK, Bertozzi S, Bloom BR, Jha P (2017). HIV/AIDS comorbidities: impact on cancer, noncommunicable diseases, and reproductive health. Major infectious diseases.

[44] Ministério da Saúde (BR). Secretaria de Vigilância em Saúde (2017). Nota Informativa nº 384/2016: mudanças no calendário nacional de vacinação para o ano de 2017.

[45] Tudor Car L, van-Velthoven MH, Brusamento S, Elmoniry H, Car J, Majeed A (2011). Integrating prevention of mother-to-child HIV transmission (PMTCT) programs with other health services for preventing HIV infection and improving HIV outcomes in developing countries. Cochrane Database Syst Rev.

[46] Drake AL, Wagner A, Richardson B, John-Stewart G (2014). Incident HIV during pregnancy and postpartum and risk of mother-to-child HIV transmission: a systematic review and meta-analysis. PLoS Med.

[47] Ministério da Saúde (BR). Secretaria de Vigilância em Saúde. Departamento de Vigilância, Prevenção e Controle das Infecções Sexualmente Transmissíveis, do HIV/Aids e das Hepatites Virais (2018). Protocolo clínico e diretrizes terapêuticas para profilaxia pré-exposição (PrEP) de risco à infecção pelo HIV.

[48] Brasil. Presidência da República. Casa Civil (2011). Lei nº 12.527, de 18 de novembro de 2011. Regula o acesso a informações previsto no inciso XXXIII do art. 5º, no inciso II do § 3º do art. 37 e no § 2º do art. 216 da Constituição Federal; altera a Lei nº 8.112, de 11 de dezembro de 1990; revoga a Lei nº 11.111, de 5 de maio de 2005, e dispositivos da Lei nº 8.159, de 8 de janeiro de 1991; e dá outras providências. Diário Oficial da União.

[49] Brasil. Ministério da Saúde. Secretaria de Vigilância em Saúde (2014). Portaria MS/GMnº 1.271, de 6 junho de 2014. Define a Lista Nacional de Notificação Compulsória de doenças, agravos e eventos de saúde pública nos serviços de saúde públicos e privados em todo o território nacional, nos termos do anexo, e dá outras providências. Diário Oficial da União.

[50] World Health Organization - WHO (2017). Guidelines for treatment of drug-susceptible tuberculosis and patient care, 2017 update.

[51] Clement ME, Okeke NL, Hicks CB (2014). Treatment of syphilis: a systematic review. JAMA.

[52] Peeling RW, Mabey D, Kamb ML, Chen XS, Radolf JD, Benzaken AS (2017). Syphilis. Nat Rev Dis Primers.

[53] Ministério da Saúde (BR). Secretaria de Vigilância em Saúde. Departamento de Doenças de Condições Crônicas e Infecções Sexualmente Transmissíveis (2020). Protocolo clínico e diretrizes terapêuticas para atenção integral às pessoas com infecções sexualmente transmissíveis (IST).

[54] Freitas MA, Miranda AE, Pascom ARP, Oliveira SB, Mesquita F, Ford N (2016). Antiretroviral therapy status among people who died of AIDS-related causes from 2009 to 2013 in Brazil: a population-based study. Trop MedInt Health.

